# The Benefits and Limitations of Evidence-based Practice in Osteopathy

**DOI:** 10.7759/cureus.6093

**Published:** 2019-11-07

**Authors:** Bruno Bordoni

**Affiliations:** 1 Cardiology, Foundation Don Carlo Gnocchi, Milan, ITA

**Keywords:** oteopathic, evidence-based practice, evidence-based medicine, cochrane, fascia

## Abstract

Evidence-based practice (EBP) arises from evidence-based medicine (EBM). The latter represents a movement of thought born in the second half of the 19th century, while the EBP is born since the new millennium, represented by different scientific figures and professional associations. The EBP is the research for the best practical and clinical strategies, with the ultimate goal of determining guidelines. The improvement of manual osteopathic practice derives from the balanced mix of scientific research, operator experience and patient experience. The text reviews the benefits and limitations of EBP in the osteopathic field. We must remember that knowledge, both theoretical and practical, is always evolving and we must not stop at what appears to be a dogma. Science and knowledge are always evolving, and hence, we must always study and update ourselves.

## Introduction and background

The evidence-based practice (EBP) arises from evidence-based medicine (EBM). The latter represents a movement of thought born in the second half of the 19th century, whose principles have been distorted over time [1]. Currently, it is believed that EBM is a pragmatic clinical choice of evaluation and therapeutic approach, faithfully reflecting what is written in the scientific literature. In reality, the cornerstones on which the EBM is based are to equate the importance of existing literature with the experience of the clinician and the patient's experience with respect to the care received [1].

Physicians David Sackett and Gordon Guyatt have laid the modern foundations of this movement born in the 1950s and 1960s, through various articles published since 1981 (in collaboration with Brian Haynes, Peter Tugwell, and Victor Neufeld), seeking the applicability of the data obtained based on the patient's need [2]. Guyatt coined the term EBM only in 1990, to make this concept (acronym) official in 1991 [2]. A Canadian and American collaboration (International EBM Working Group) was created to try to improve the medical approach with this new scientific idea. From this collaboration was born JAMA User's Guide to the Medical Literature, until obtaining a general approval in the scientific field [2].

In 1996, Sackett, given the difficulty in reconciling the daily clinic with the ever-evolving scientific knowledge, wrote an article to explain, once again, the real meaning of EBM: “The practice of evidence-based medicine means integrating individual clinical expertise with the best available external clinical evidence from systematic research. By individual clinical expertise, we mean the proficiency and judgment that individual clinicians acquire through clinical experience and clinical practice…..and in the more thoughtful identification and compassionate use of individual patients' predicaments, rights, and preferences in making clinical decisions about their care.” [3]. The article specifically explained that the final decision to orient the physician towards the most appropriate treatment must be based on the contemporary use of the clinician's experience, the patient's experience and will and scientific evidence. The EBM serves to push the clinician to update and apply the best scientific knowledge in patient care, making autonomous decisions. It becomes a mistake to seek clinical decisions using one of the components alone [2-3].

In 2008, Montori and Guyatt wrote an article to emphasize how, in addition to the importance of scientific evidence and the clinician's experience, the patient's experience of treatment has the same importance in the final curative decision [4]. EBP is an evolution from the EBM, starting from the new millennium, represented by different scientific figures and professional associations. EBP is the research for the best practical and clinical strategies, with the ultimate goal of determining guidelines [5]. This new clinical approach is based on six basic steps. The first concept of EBP focuses on patient care (patient-centred care); the second and third concepts are given by the achievement of the therapeutic solution through a multidisciplinary work and considering the scientific literature; the fourth step is aimed at improving therapeutic quality, which is linked to the fifth point, namely, the safety of this clinical evolution, to end with the latest concept based on the use of technology to keep up to date [6].

There are some assessment tools to understand if there is an acceptable level of knowledge that coincides with clinical quality, such as the Berlin questionnaire (self-administered test on 15-item), the Fresno tool (a test created with clinical scenarios with open-ended questions) and the Assessing Competency in Evidence-based medicine (ACE) tool (a more complex test where the attitude is also evaluated) [6]. The EBP reflects the nature of EBM, integrating the different experiences (clinical and patient) in a multidisciplinary union, using the best technologies and scientific research [6-7]. The text reviews the benefits and limitations of EBP in the osteopathic field.

## Review

The benefits of EBP in osteopathy

Osteopathy considers the systemic relationships of the human body, during both evaluation and manual practice [8]. Like all scientific disciplines that wish to have a recognized role in the treatment of patients, EBP is definitely a strategy to implement the importance of the manual approach and to have a suitable tool for a comparison with other health figures. Osteopathy has clinical and practical information that can be extracted from the literature, and this facilitates the work of the operator, with positive patient response. One of the reference points of EBP is the Cochrane review data collection and the metanalytic review studies. EBP is based on the collection and analysis of randomized controlled trials, where the results (positive or negative) automatically become a practical guide for clinicians [9]. In the structural field (skeletal muscle), we can affirm that the vertebral manipulations and at the level of pelvic-related joints, through direct techniques such as high-velocity low amplitude thrust (HVLA), are considered effective in reducing perceived pain in patients with low back pain (subacute and chronic), neck pain, prophylaxis of migraine and cervicogenic headache [10].

In a cardiovascular field, one of my recent studies has demonstrated how osteopathy with indirect techniques for patients undergoing cardiac surgery, not only decreases the pain perceived by the patient, but the general clinical picture improves in early times and with a more rapid discharge [11]. We know that osteopathic treatment is effective in preterm children, with a reduction in the length of stay and without any adverse effects or clinical complications [12]. In patients with knee osteoarthritis, a randomized trial showed that osteopathy is able to reduce pain perceived, with a superior joint functional improvement, compared to the usual rehabilitation [13]. The use of osteopathy to reduce pain and improve the functional status of pregnant women (from the third month of pregnancy) works, without side effects of any kind [14]. A multicenter, randomized controlled study shows that the manual osteopathic approach for older adults hospitalized for pneumonia is able to reduce hospitalization time and reduce mortality [15]. We can say that an osteopathic craniosacral treatment decreases the intensity of perceived pain in patients with chronic low back pain; in addition, systemic parameters such as blood oxygenation, blood pressure values and blood potassium and magnesium values are improved [16].

Another randomized trial with a follow-up of five years demonstrates how a manual osteopathic approach can significantly improve the symptoms of patients with chronic prostatitis with chronic pelvic pain [17]. In patients with diabetes mellitus and suffering from low-back pain, a study showed that the osteopathic approach significantly decreased perceived pain, with systemic reduction of inflammatory cytokines [18]. Osteopathy is able to improve some symptoms in patients with irritable bowel syndrome (IBS), such as reducing the sensation of abdominal pain and decreasing rectal sensitivity [19]. EBP allows us to affirm that osteopathic treatment acts positively on several fronts, such as the structure and somatic pain, it allows an earlier hospital discharge in neonatal settings, in the cardiology field and in the elderly with pneumonia. We can say that in pregnant women, since the third trimester, osteopathy improves the quality of life of the woman in labor without any side effects. We have interesting evidence for osteopathic efficacy in prostatic and intestinal problems and in the reduction of systemic inflammatory cytokines.

The limitations of EBP in osteopathy

The use of EBP in the osteopathic field is still a practice not followed in a decisive manner [20-21]. The reasons may be different. For example, scientific evidence in clinical practice is not a constraint, but a direction: “External clinical evidence can inform, but can never replace, individual clinical expertise, and it is this expertise that decides whether the external evidence applies to the individual patient at all, and if so, how it should be integrated into a clinical decision.” [22]. EBP does not always exist to be applied to the specific case, or, in particular, that notion is not suited to the type of dysfunction. A lot of EBP addresses an area, a pathology but in everyday life, patients who turn to the osteopath have different overlapping disorders [23]. Currently, literature has become "dictatorial", when even the principles that led the epidemiologist Archie Cochrane to devise the Cochrane review have been arbitrarily interpreted in the last 20 years [9]. Dr Cochrane gave less importance to randomized studies and aimed to give greater emphasis to care and equality between different clinical and cultural sub-populations [9].

EBP is an important resource for a manual approach, a tool that must be used based on the patient's needs and weighing the scientific information with respect to the experience of the operator and patient. In fact, the manual best practices are based on other more loose principles: “Identify and develop clinical models that integrate evidence-based nonpharmacologic therapies…. Develop, optimize, incentivize and coordinate care across disciplines with nondiscriminatory access to evidence-based nonpharmacologic therapies, as a stand-alone first line of care and as an essential part of comprehensive care.” [10]. It is this bad interpretation of the meaning of EBP that probably creates a gap between the osteopath and the more frequent use of scientific information. Taking up an article by another author, we should move from EBP to EBOM, that is, evidence-based osteopathic medicine [24]. Once again it is criticized that the result of randomized research is more important than the subjectivity of the patient [24]. According to other authors, the clinical results of the trials omit important details, which make it difficult to apply the advantages brought by research in the practical field (psychological aspect, individuality, co-morbidity, and more) [2]. If the base from which a trial starts is of low quality or is based on incorrect assumptions, the result will necessarily be of low quality or with errors (type 1 or type 2 error); it may happen that a metanalytic review highlights quality errors on trials considered to be of high quality [2]. This can lead the clinician to choose low-quality randomized trials, compared to observational studies, the latter being considered less important due to the incorrect imprinting given by EBP [2]. Some EBP trials report negative results, but simply because the data on which the conclusions are based are poor or cannot be compared with other studies [25].

Implications or relevance of EBP in osteopathy

How can we apply scientific information in the osteopathic field? Probably, we must remember that the results in literature do not include all types of patients and that even simple case reports are important to understand and improve the daily osteopathic practice [26-27]. The same is true for revisions, not necessarily systematic ones, which highlight some practical aspects from different studies, giving food for thought or highlighting areas of knowledge that are lacking [28-29]. Articles that discuss hypotheses of treatment are just as important to get ideas to put into practice, or to compare the information presented with the osteopath's daily clinic [30-31]. For example, one of my articles on the diaphragm has shown that the muscle is not innervated by the intercostal nerves, but by the phrenic nerve and the vagus nerve [32]. This banishes an osteopathic approach that involves the intercostal nerves and associated structures (ribs, intercostal muscles, and others); rather, the manual approach must also be re-evaluated towards the vagus nerve to improve the exhaustive action of the diaphragm muscle. Another example is to re-evaluate liquid techniques in the osteopathic field, thanks to articles that demonstrate how body fluids (blood, lymph) are part of the fascial system, and creating space for further research [33-34]. Again, revision texts can help the daily osteopathic clinical practice, reaffirming that bone tissue is an integral part of the fascial continuum, re-evaluating bone techniques (intraosseous lesions, and more) [35].

To conclude with examples of how a revision can be useful to change the manual approach, other texts highlight that the cranial articulation between the sphenoid bone and the occipital bone from adolescence merges, and all the meaning of cranial primary breathing must change, as well as practice [36-37]. EBP is all that scientific literature reports; the osteopathic clinician must be able to extrapolate the information that can be useful to improve the salutogenic result of the patient, in light of his/her own experience and the subjective need of the person under his/her own care. Literature is not an absolute constraint or a dogma, but a constant stimulus for updating.

## Conclusions

The EBP derives from the EBM, currents of thought with the ultimate goal of improving the osteopathic clinical decision. This can happen, thanks to a balanced mix of scientific research and operator experience and the patient's experience. Literature in recent decades has misrepresented the meaning of EBP/EBM, creating hierarchies of different studies, putting randomized controlled research on top of this erroneous evaluation, which is considered the only source of error-free information. In reality, the tendency of this vision of literature has brought to light the limits, or rather, the not easy applicability of the results coming from these researches and wrongly obscuring the validity of other types of articles. The osteopathic practice must be able to take advantage of all the scientific information available in the literature, as the manual clinic is full of facets not always found in the trials.
